# Development research on an AI English learning support system to facilitate learner-generated-context-based learning

**DOI:** 10.1007/s11423-022-10172-2

**Published:** 2022-12-12

**Authors:** Donghwa Lee, Hong-hyeon Kim, Seok-Hyun Sung

**Affiliations:** 1 Institute of Education, UCL, 20 Bedford Way, London, WC1H 0AL UK; 2 ZEROxFLOW, 16F FRONT1 Bldg, 122 Mapo-Daero, Mapo-Gu, Seoul, Korea

**Keywords:** Artificial intelligence, Learner-generated context, Learner-generated content, Intelligent computer-assisted language learning, Development research

## Abstract

For decades, AI applications in education (AIEd) have shown how AI can contribute to education. However, a challenge remains: how AIEd, guided by educational knowledge, can be made to meet specific needs in education, specifically in supporting learners’ autonomous learning. To address this challenge, we demonstrate the process of developing an AI-applied system that can assist learners in studying autonomously. Guided by a Learner-Generated Context (LGC) framework and development research methodology (Richey and Klein in J Comput High Educ 16(2):23–38, https://doi.org/10.1007/BF02961473, 2005), we define a form of learning called “LGC-based learning,” setting specific study objectives in the design, development, and testing of an AI-based system that can facilitate Korean students’ LGC-based English language learning experience. The new system is developed based on three design principles derived from the literature review. We then recruit three Korean secondary-school students with different educational backgrounds and illustrate and analyze their English learning experiences using the system. Following this analysis, we discuss how the AI-based system facilitates LGC-based learning and further issues to be considered for future research.

## Introduction

Artificial intelligence (AI) consists of “intelligent computer systems or intelligent agents with human features, such as the ability to memorize knowledge, to perceive and manipulate their environment in a similar way as humans, and to understand human natural language” (Zawacki-Richter et al., 2019, p. 10). Education researchers have studied AI applications in education (AIEd) for decades to enrich learning and teaching activities (Luckin et al., 2016). Given the shift from in-person to remote schooling brought about by the COVID-19 pandemic (Pantelimon et al., 2021), scholars have focused on creating various AIEd applications such as virtual assistants for teaching course content, automated grading and feedback, and learner-tracking systems (Dignum, 2021; Zawacki-Richter et al., 2019).

However, studies that critically reflect on past AIEd efforts present a challenge: AI applications should be guided by pedagogical theory or learning science to induce meaningful changes in teaching and learning (Luckin & Cukurova, 2019; Zawacki-Richter et al., 2019). Here, “*meaningful changes*” means those that incorporate not only technical but also pedagogical, sociocultural, and ethical factors. Moreover, AI applications should meet the needs of specific educational domains, goals, or activities (Zhai et al., 2021).

Among these challenges of AIEd, one specific need arose during the COVID-19 pandemic. Without adequate preparation, students were forced to study independently while being physically away from educational institutions and influenced by the digitization of educational processes (Wismaningrum et al., 2020). Students increasingly need digital assistance in studying autonomously and flexibly and in adapting themselves to complex social situations (Ariebowo, 2021; Hargreaves, 2021; Maru et al., 2021; Wismaningrum et al., 2020).

Most previous computer-aided instruction and virtual education environments have been restricted to classical classroom settings, but AI offers possibilities for new forms of educational support (Loeckx, 2016). Some recent studies have demonstrated how AI-informed systems help learners learn autonomously in accordance with their self-determined goals (e.g., Ahmad & Ghapar, 2019; Inyega & Inyega, 2020). However, such studies remain insufficient and are mainly conducted in higher education settings. More AIEd research efforts are needed to support students in different contexts and thus contribute meaningful changes in the field.

In an attempt to address this particular challenge of AIEd, we present a development study that examines the process of developing an AI-based system capable of assisting learners in autonomous study. This development study was guided by an educational technological framework called a Learner-Generated Context (LGC). Many elements must be considered when supporting learners’ autonomous and independent learning (Hargreaves, 2021). LGC as a framework helps researchers conceptually capture what form of learning and relevant elements to pursue in assisting learners to study autonomously and in designing and applying technologies accordingly. Another consideration is the recent criticism that AIEd is being used to monitor learners more than is necessary (Zawacki-Richter et al., 2019). Following the LGC framework, the design of our AI application defines the position of learners in their interactions with digital technology without being needlessly invasive. In terms of methodology, our study follows Richey and Klein (2005).

## The LGC framework

### LGC-based learning

The LGC framework was developed based on the following views of modern society and the role of education. First, the complexity of knowledge construction in modern society makes it difficult for individual learners to understand knowledge by passively following curricula and textbooks. Thus, education should help learners explore different contexts and resources and construct knowledge autonomously (Pachler et al., 2009). Second, with the development of technology—including mobile and social networking technologies and services (e.g., Facebook, Flickr)—people have grown accustomed to user-generated content and activities at anytime and anywhere of their choosing. If digital technologies are used appropriately, learners can become active creators who forge their own learning paths (Luckin et al., 2011).

Based on these views, LGC presents a form of learning through which learners become the creators of their learning contexts and construct knowledge autonomously by interacting with or creating resources (e.g., people, technology, and learning contents) with self-defined learning goals in various environments (Luckin et al., 2007; Sharpe, 2010). In this study, we use the term “LGC-based learning” to refer to this form of learning.

### Facilitation of LGC-based learning

LGC as a framework also illustrates concepts, conditions, and strategies for facilitating LGC-based learning through the design of digital technology. Here, facilitating learning means using digital tools as “a catalyst” for pedagogical “change” (Kukulska‐Hulme, 2010, p. 181). The core idea of facilitating LGC-based learning is to design and apply digital technology in a manner that assists learners to form a “learner-generated context” and learn based on this context. The application of this idea can be categorized into three: designing technology for learners’ contexts, personalized learning support, and assisting learners’ interactions with resources.

#### Designing technology for learners’ contexts

In designing instruction models or learning tools, designers should consider the context in which learning experiences form (Luckin et al., 2011, 2013); following the LGC framework’s conceptual assumptions about learning, specifically that learning is constructed across a continuum of contexts in which learners interact with other individuals, groups, resources, and events from multiple physical spaces and times (Luckin et al., 2011). Depending on how a context is formed through the use of digital technologies, it encourages learners to build their knowledge and craft meaningful connections with society, providing them with deep and broad learning experiences (Brown, 2003).

There are two types of learning contexts defined by who controls educational activities; the first involves externally delivered resources to learners who must behave in certain ways to learn (Pachler et al., 2010). The second forms naturally as individual learners pursue their self-defined learning purposes and create or use learning content that fits their purposes while interacting with various resources and environments (Cochrane & Narayan, 2011; Luckin, 2010; Luckin et al., 2011). The latter is what Luckin et al. (2011) called a learner-generated context, which the LGC framework assumes to be the preferred context for LGC-based learning. This means giving learners the freedom to generate and control their own learning context while discouraging the unilateral presentation of organizational imperatives or contexts to direct learners' choices.

#### Personalized learning support

LGC addresses that learners naturally generate their own learning contexts in the process of determining or creating the elements of their learning such as goals, contents, places, and strategies. It is important to offer personalized learning support that guides learners to determine what, where, when, and with whom their learning takes place (Cochrane & Bateman, 2011; Narayan & Herrington, 2014; Narayan et al., 2019). First, learners should be guided to use learner-generated content so that they can explore content generated by other learners that they can then use for their goals (Lee & McLoughlin, 2007). Meanwhile, they should be able to create or transform their own learning content (Luckin et al., 2011). Through this, learners can perceive themselves as creators who create their knowledge and study opportunities and share them with other learners. Second, learners must be able to conduct their learning anytime and anywhere using mobile technology (Cochrane & Bateman, 2011).

Third, rather than inducing learners to do everything independently, learning aids should be provided flexibly in accordance with each individual learner’s context. Luckin et al. (2011) termed this type of personalized learning assistance the “pedagogy–andragogy–heutagogy (PAH) continuum.” Based on the learner’s context, digital assistance can be provided that helps learners understand curricular subject knowledge (pedagogy) or enables them to negotiate with instructors or experts to set their own learning path (andragogy), thereby allowing them to develop “the understanding that one is empowered to look at the learning context afresh and take decisions in that context” (heutagogy) (Luckin et al., 2011, p. 78). For example, given various learning strategies, learners may try classic strategies such as teacher lectures or problem-solving activities to deepen their understanding of curricular subject knowledge. Alternatively, learners may prefer to interact with learning partners when deciding what to do.

#### Assisting learners’ interactions with resources

Digital assistance should encourage learners to not only stay within their chosen learning contexts but also make connections with other contexts (Aguayo et al., 2017). From the continuous interactions between learners and resources, learner-generated contexts can be continuously shaped and extended in the form of social networks (Narayan & Herrington, 2014). Therefore, individual learners can acknowledge the wider world and further develop their perspectives, interests, and knowledge.

For this, the LGC framework suggests using open educational resources (OERs)—ready-made learning content—and developing an open online platform where learners can explore, curate, and share various learning resources as social networking-based learner communities. On such a platform, learners can make connections with other learners, open resources, and learning contexts, thereby continuously expanding their experiences and knowledge (Blaschke & Hase, 2016; Luckin et al., 2011).

### Previous LGC research and this study’s goal

Previous LGC studies have demonstrated various types of assistances that can facilitate LGC-based learning—particularly support in personalized learning activities, resource exploration, and content creation—that include applying social networking tools (e.g., blogs or Facebook) and mobile devices (see, e.g., Aguayo et al., 2017; Cochrane et al., 2012; Cook, 2010; McLoughlin & Lee, 2008; Narayan et al., 2012). These studies mainly focused on instruction models rather than developing a learning support system because they assumed the presence of human instructors capable of providing timely feedback and encouragement. However, current AI technology has developed to where human roles can be replaced to some extent. For example, learners can interact with AI-applied tools even in the absence of human instructors, thereby minimizing their fear of potential failure and learning with confidence. This advantage has been demonstrated in second-language education research (e.g., Fryer et al., 2020; Woo & Choi, 2021). Thus, we expect that AI-based systems can further enrich the LGC-based learning experience depending on how LGC-informed design and AI technologies are applied together.

This study’s goal is to develop an AI-based system that can facilitate LGC-based learning experiences. The results of this study present an AI-applied tool that can help meet the needs of educational fields for LGC-based learning and provide a reference for AIEd research and practice seeking similar goals. We expect that this study can help promote the understanding and practice of incorporating educational knowledge into the design of AI technology for learners’ autonomous and independent learning. In pursuit of this goal, we applied Richey and Klein (2005)’s development research method because it offered a systematic and focused way to achieve our goal.

## Methodology

### Development research

According to Richey and Klein (2005), development research for an educational tool entails defining the problem and context on which to focus, the literature review and procedures for designing, developing, and evaluating the new tool. The main consideration is that the researcher should not simply pursue developing the new tool itself but instead aim to solve problems through its development. First, the researcher determines the specific context that requires a new tool to solve a certain problem and, following this context, the specific research objective. Gaining insight from the relevant literature, the researcher then designs and develops the tool. After that, the researcher examines whether the developed tool produces an expected effect based on the data collected from field tests and identifies the tool’s improvement potential and research implications.

In setting our research objective, we situated this study in a specific context that requires an AI-based system that can facilitate LGC-based learning: English language education in South Korea.

### Context and objective for developing the system

Korea has been reported to have the highest level of participation in distance education among Organization for Economic Co-operation and Development (OECD) member countries, as well as a high proportion of youths with basic skills for technology-rich environments (OECD, 2019). Despite this, online English education in Korea does not provide the most suitable environment or tools for realizing LGC-based learning. First, English education in Korea is characterized by a context that emphasizes academic achievements and exams. Under this context, most Korean students—especially secondary-school students—follow desired processes defined by policymakers or experts with reference to the national curriculum and college entrance exams (Chang, 2006; Jeon, 2010; Kim & Won, 2019). This context reduces the range of knowledge of the English language that learners can develop and does not reflect the complexity of individual learners’ contexts. Second, the pandemic has made it apparent that Korean students are struggling with autonomous learning (Korea Education & Research Information Service, 2020). While the number of online courses has increased, many Korean students have reportedly failed to study English independently (Kim et al., 2020). However, according to Oh (2022), Korean students will likely participate in English learning more actively if provided with English study opportunities to pursue their own contexts, goals, interests, and learning strategies.

In the current literature, we found no studies that apply the LGC framework to English education in Korea. However, to assist Korean students’ autonomous learning in English speaking and writing, some scholars have investigated AI applications such as conversational AI chatbots, AI speakers, machine translations, and automated grammar checkers (e.g., Hyun & Im, 2019; Kim et al., 2019; Lee, 2020; Lee & Briggs, 2021; Park, 2019; Park & Yang, 2020). These researchers have provided ways to help learners practice speaking and writing in English without the help of lecturers, but their efforts do not provide opportunities for learners to explore or create the most effective learning strategies and content for themselves. Korean learners still need further AI support to fully experience LGC-based English language learning. Thus, this study’s objective is to design, develop, and test such a support system.

### Research questions and procedures

The following questions were set to guide this development research: First, what design principles define the necessary functions of an AI-based English language learning support system that can facilitate LGC-based learning? Second, how are the design principles realized in the development process? Third, does the developed system catalyze LGC-based learning experiences?

For the first question, we developed three design principles for the new system based on the literature review and investigations of applicable AI technology. For the second question, we developed and described the new AI web-based system in accordance with the design principles. For the third question, we conducted a field test to validate the system by recruiting three Korean secondary-school students and analyzing the narratives of their experiences using the system. The narratives were analyzed following the evaluation criteria, which were consistent with our design principles and qualitative data analysis strategies.

After these procedures, we critically reviewed our process and prepared this report on outcomes and improvement points for the system and the study’s implications.

## Construction of design principles

We reviewed the literature on LGC and relevant concepts, such as self-determined learning, that correspond to the characteristics of LGC-based learning. Considering the PAH continuum idea, we also reviewed the literature on second-language learning strategies applicable to LGC-informed AI technology design. Three design principles of the new system were derived from our findings.

### Design principle one

In the design process, learners were regarded as creators capable of generating their own contexts and studying autonomously based on such contexts, through personalized learning assistance and continuous interactions with resources.

### Design principle two

A system should provide learners with personalized support in determining or creating the elements that make up their learning context (e.g., learning content, plan, and strategies). We consider the following functionalities as specifically optimal for a new AI-based system that facilitates LGC-based English language learning:

- enable learners to pursue learning anytime anywhere through mobile and web-based learning experiences (Djoub, 2016; Lai, 2019; Palalas & Wark, 2020; Vavoula & Sharples, 2002).
- use learner-generated content. Rather than offering pre-selected knowledge as in traditional textbooks, learners must be allowed to explore potential learning materials, such as photos or video clips, or create learning content on their own (Cook, 2010; Luckin et al., 2011; McLoughlin & Lee, 2008). Particularly in language education, learner-generated content can lead to more active learning engagement than teacher-provided learning content (Lambert et al., 2017).
- use OERs or resources from online media platforms, such as YouTube, blogs, or Internet forums, to give learners flexibility in deciding what and how to learn and create knowledge (Cronin, 2017; Duffy & Bruns, 2006; Rahimi et al., 2015).
- assist learners in determining which learning strategies are right for them. In second-language learning, the system might provide multisensory learning strategies, allowing learners to choose the best learning strategy for them: through sight (e.g., highlighting keywords, repeatedly seeing vocabulary on flashcards), auditory stimuli (e.g., reading aloud, listening to audio online), or kinesthetic activities (e.g., physical movement, such as typing) (Juřičková, 2013).
- apply mobile, multimedia, and natural language processing (analyzing and representing human language) technologies that enable learners to use learning content from different types of resources and use diverse senses in learning activities (Cook, 2010; Smrz, 2004; Zhang & Nunamaker, 2004). Multimedia and natural language processing technologies can express knowledge in various modes or metaphors, thereby supporting individuals in cognitively processing their sense experiences and effectively building knowledge from those experiences (Ox & Van Der Elst, 2011).
- utilize an intelligent agent (i.e., an autonomous entity that acts on an environment or user input) (Conati, 2009) to facilitate learning with no human instructors. For instance, a system can offer learners guidance for solving problems through techniques like questions, feedback, and explanations of issues (Fernández et al., 2015). An autonomous agent equipped with speech recognition can also understand a learner’s speech and give immediate feedback when the learner is practicing English speaking alone (Hyun & Im, 2019; Kim et al., 2019).

### Design principle three

To encourage learners to interact with resources and expand their contexts, an open platform of learning resources should be provided. Following previous LGC studies, we sought to make it possible to use OERs within the new system. We also tried to incorporate an open platform of resources into the system which can be accessed through a content curation tool. According to Ponerulappan (2015), this curation tool organizes and curatorially presents a broad range of e-resources so that learners can easily search, explore, and select them. It allows users to explore and share resources and interact with other users easily.

## Development of the new system

### Overall system architecture

**Fig. 1:**
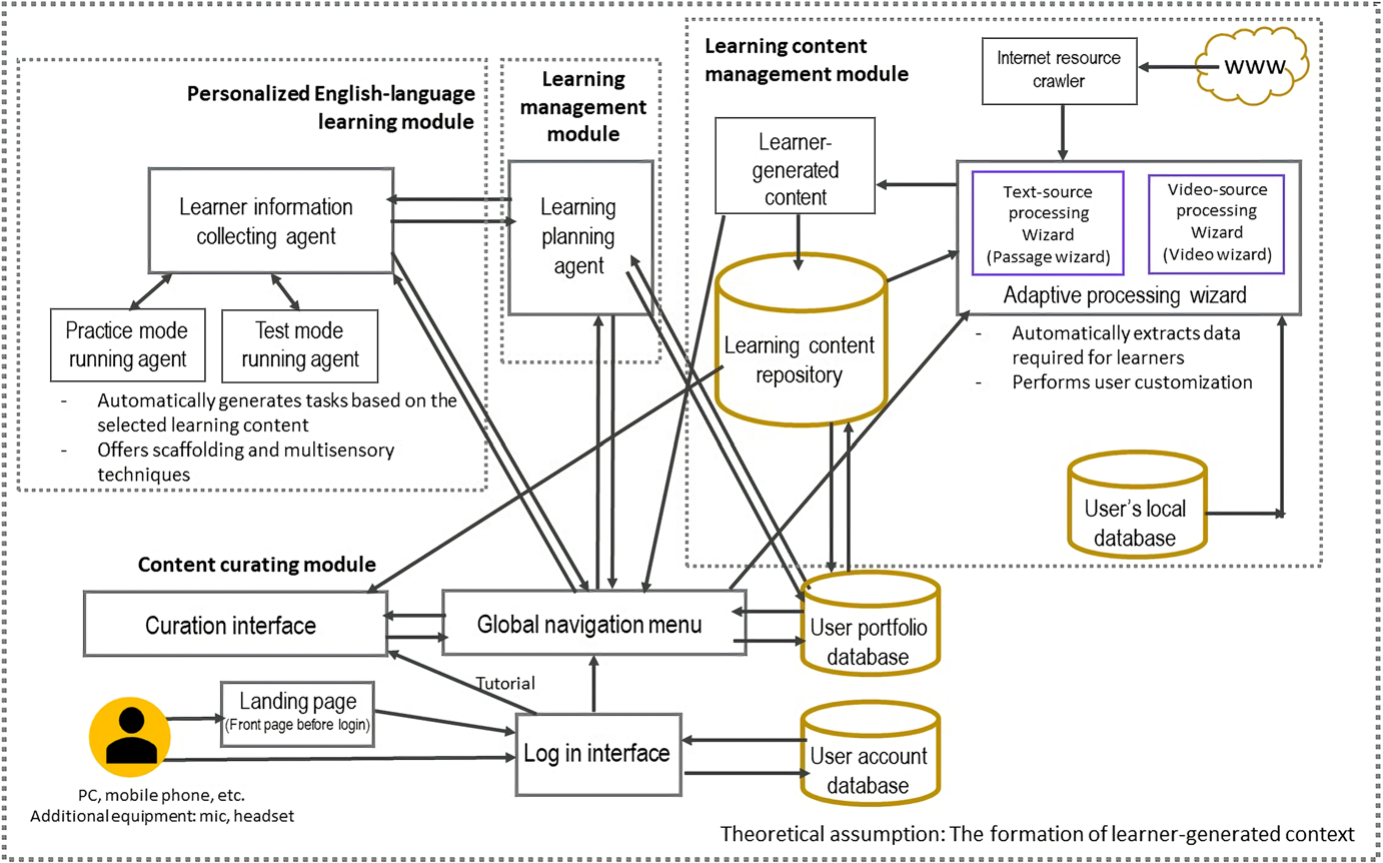
System architecture

With reference to the design principles, we created an AI web-based English learning support system that can be used in web and mobile environments. The system has four functional modules: (1) learning content management, (2) learning management, (3) personalized English language learning, and (4) content curation. Figure 1 shows the system architecture.

### Learning content management module

The learning content management module is an automated system for creating or editing digital learning content in two forms: an English vocabulary list and a sentences and quotes list. Two editors support the function of this module: a passage wizard and a video wizard.

#### Passage wizard

The passage wizard analyzes the sentence components of English texts included in various digital and analog data—such as paper books, Internet articles, and news articles—and creates English vocabulary or sentence lists based on this analysis. The lists are used as learning content within the system.

**Fig. 2:**
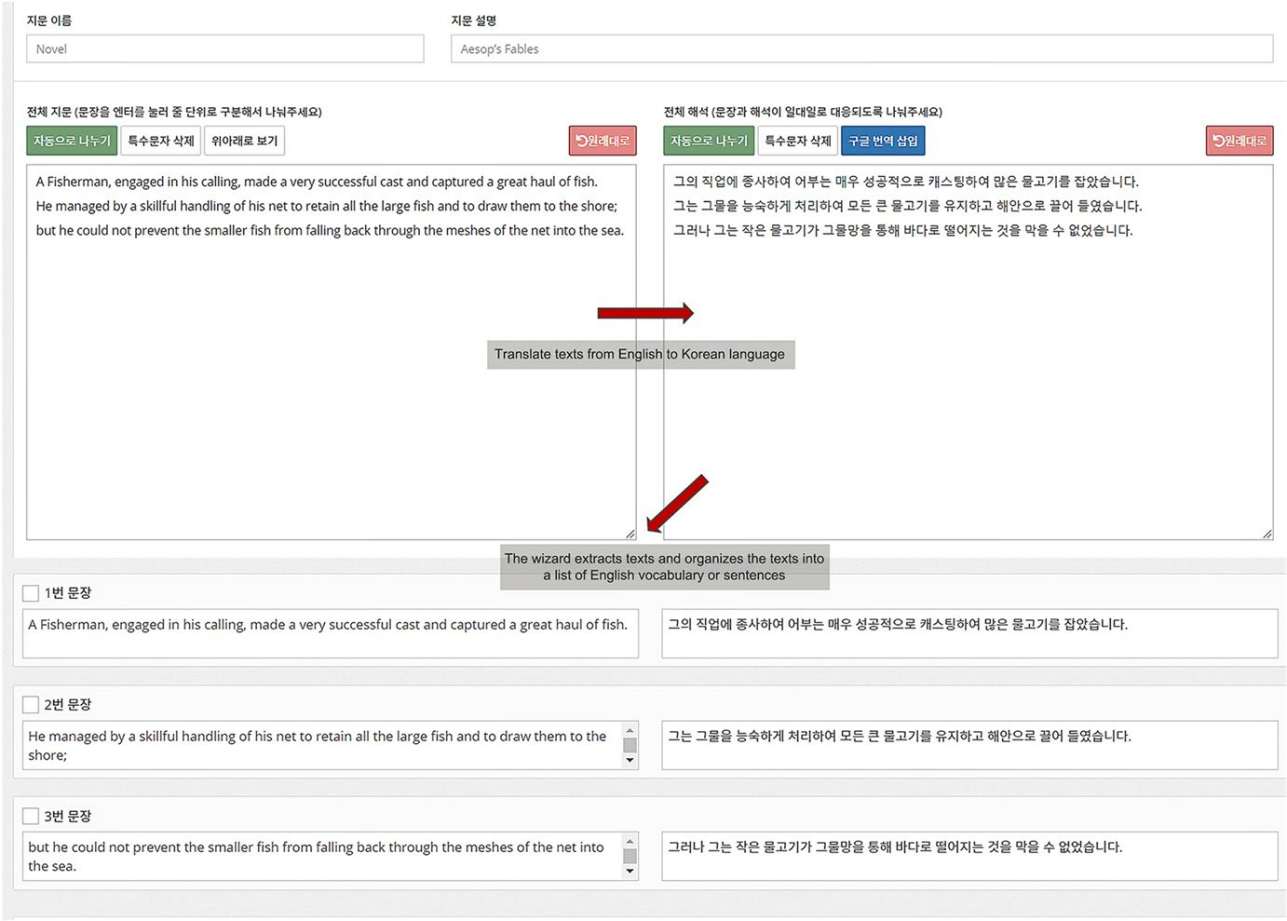
User interface for processing text-based materials into a list of vocabulary and sentences. *Note* When a learner copies and pastes English text into a text box, the wizard organizes it into a list of English vocabulary words or sentences with a Korean translation

When learners upload English textual material relevant to their current interests or needs to the passage wizard or enter text directly into the wizard’s text box (Fig. 2), the wizard analyzes and extracts text from the material and organizes it into a list of English vocabulary words or sentences. Sequentially, it adds translations and voice data to the list. These functions serve three purposes: (1) notifying learners of the meaning of vocabulary words; (2) using the voice data to provide auditory stimulation to help learners acquire the correct pronunciation in subsequent learning; and (3) saving all text and relevant data as learning content.

To implement the wizard’s functions, we used application programming interfaces (APIs) that are widely adopted in open source AI communities. First, we used the natural language syntactic analysis provided by the Google Cloud Natural Language API in our text extraction algorithms. This API enables the wizard to recognize the structure and meaning of English texts that learners enter into the wizard by identifying English sentences and their components, analyzing the relationships between them, and creating a parse tree of each sentence. Based on this analysis’ results, the wizard categorizes the sentences, or the words and idioms comprising each sentence, and presents them to the learner in the form of a list of English sentences or vocabulary words.

**Fig. 3:**
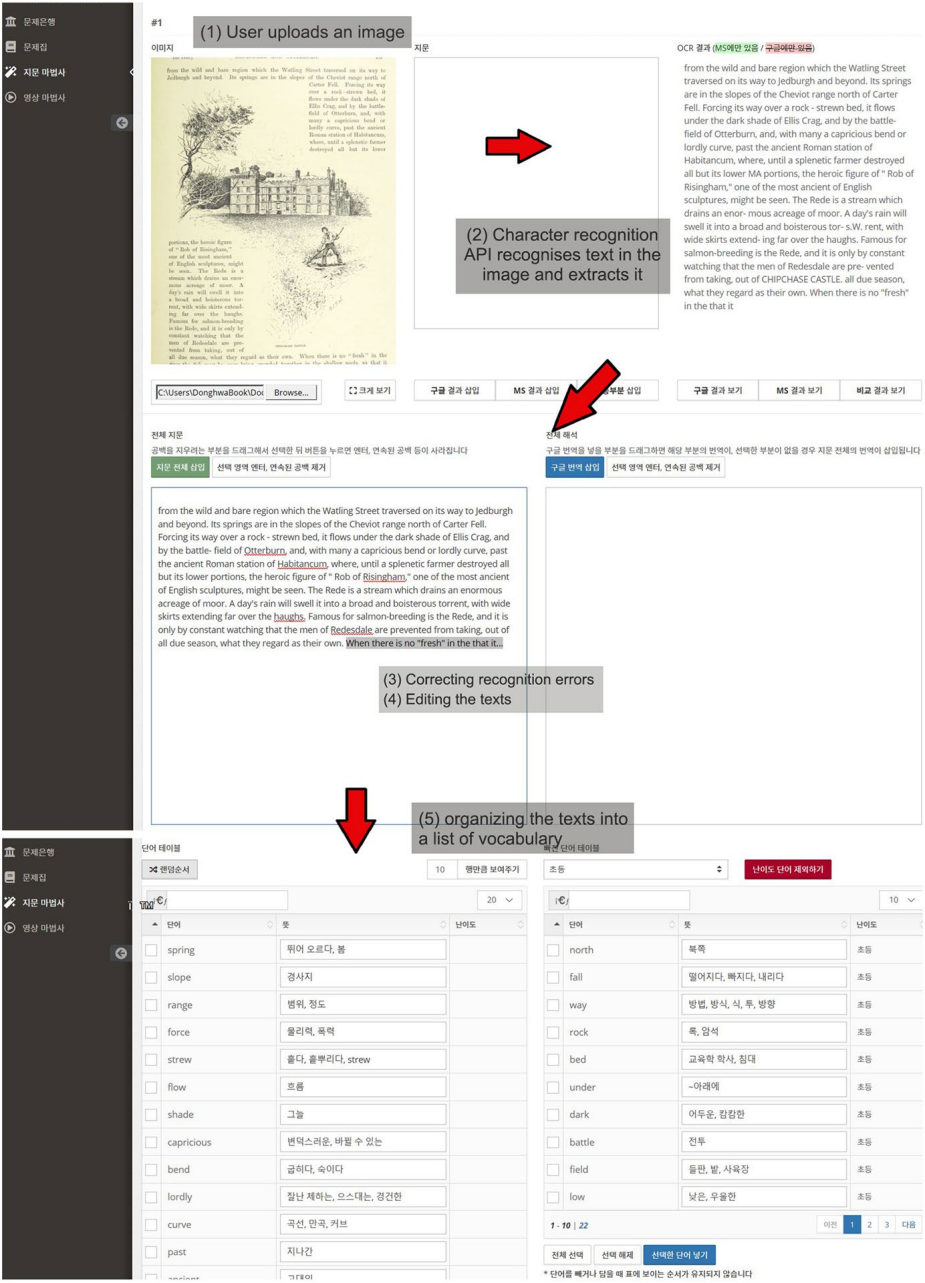
Learner interface for processing image-based materials. *Note* When a learner uploads image material containing English passages, the OCR API recognizes and extracts text in the image. The learner can check the extracted text to correct errors or remove unwanted text. Finally, the passage wizard organizes the text into a list of English vocabulary words or sentences with a Korean translation. The sample image is a copyright-free image released by the British Library on Flickr Commons for anyone to use, remix, and repurpose

Second, to generate translation and voice data for each vocabulary or sentence item in the list, we used Twinword’s Word Dictionary API and Amazon Polly, a text-to-speech converter. In addition, Google and Microsoft’s optical character recognition (OCR) APIs were built into the passage wizard to assist learners in creating learning content based on analog material. For example, when a learner takes a picture of paper material with English text and uploads it to the passage wizard via a mobile device, the OCR API recognizes text in the image, thereby enabling the natural language syntactic analysis function to work (for an example, see Fig. 3).

#### Video wizard

**Fig. 4:**
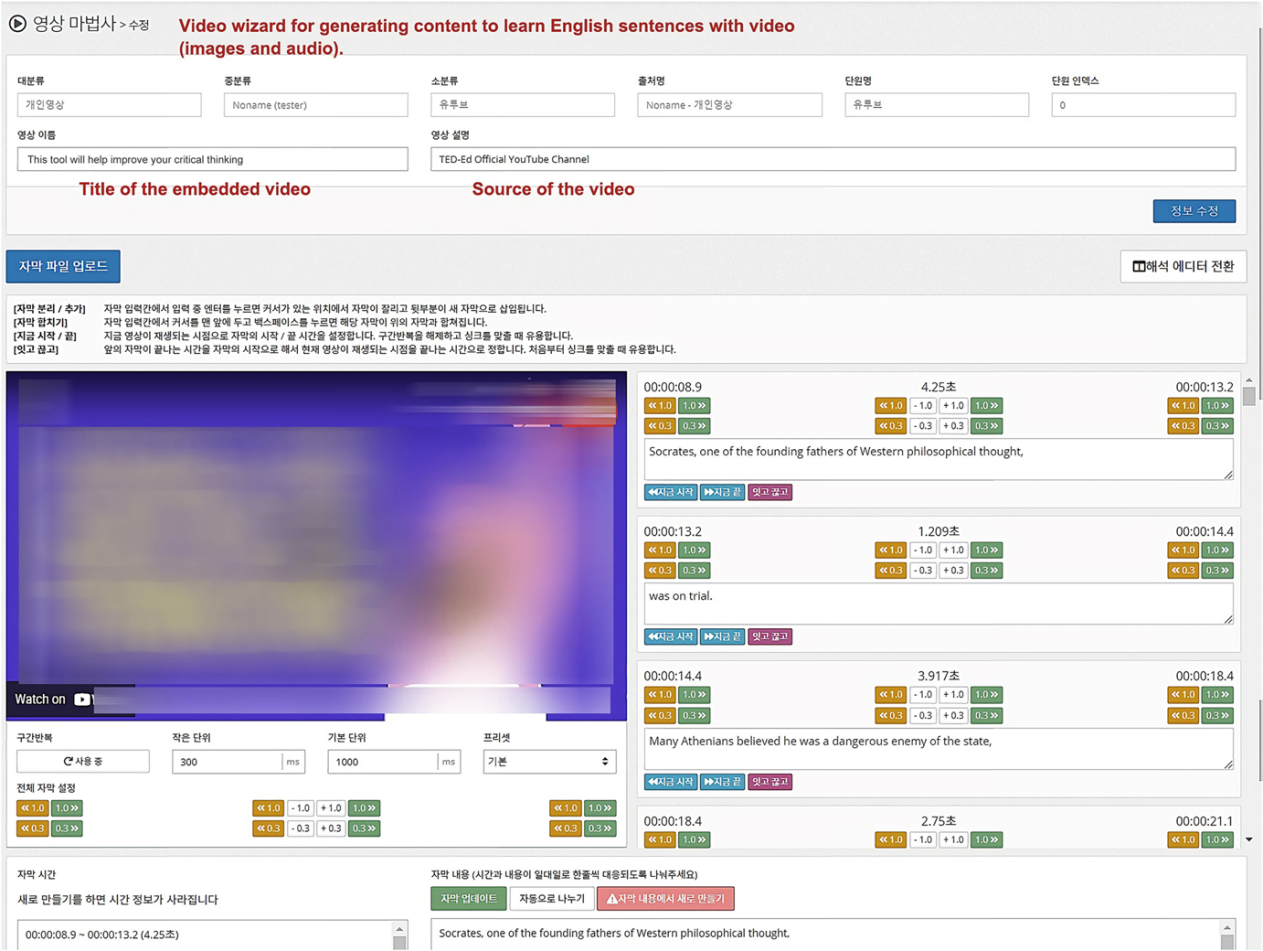
List of captions and time stamps presented by video wizard. *Note* The video wizard was designed to prevent copyright infringement, not to reproduce copies of video works. Using the video embedded code from YouTube, the wizard solely provides a caption and time selection and segment repeatability functions for learners while preserving original works. The video in the figure was from TED Edu YouTube channel (see https://youtu.be/vNDYUlxNIAA)

The video wizard was designed to analyze and repeat specific parts of a YouTube video, thereby helping students study English dialogue with the video. The expected scenario for a learner’s use of this wizard is as follows: First, the learner opens the wizard and inserts the embedded URL of a specific YouTube video clip containing English dialogue and captions. Anything that a learner likes can serve as learning content—for example, official YouTube videos that are relevant to his or her favorite movies, games, and music videos. When the video is embedded on the wizard, the wizard identifies scenes with dialogue. It then syncs dialogue with captions, time-stamps the scenes, and presents the captions and corresponding audio with time stamps (Fig. 4).

Next, the learner marks the times of the sections of the video that contain specific English sentences that they wish to study. Then, the wizard stores the marked sections of the video, the corresponding caption, and video data as a set with the embedded video. This set of data is named as video-based learning content in the system.

#### After generating learning content

Learning content created through the passage wizard or video wizard is uploaded to the system’s learning content repository as well as the user’s personal database and is stored as data that can be shared with other users.

### Learning management module

**Fig. 5:**
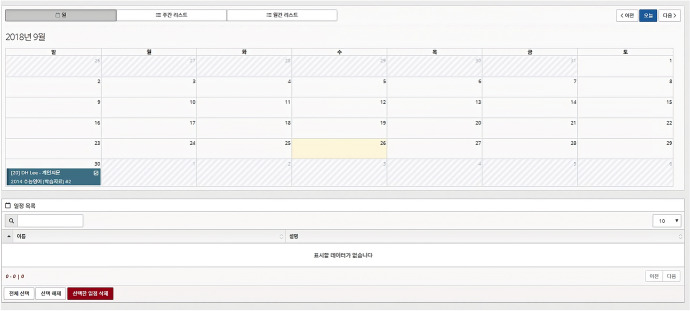
UI for scheduling

Once learning content is available, learners can arrange their learning schedules through the calendar-shaped user interface (UI) of the learning management module (Fig. 5).

When a user selects a certain date on the calendar-shaped UI, a window opens to choose a mode of learning: (1) words and phrases learning mode or (2) sentences learning mode. When the learner chooses a mode, the system opens a new window and loads the stored learning content from the system repository or the user’s personal portfolio database. The learner then chooses one piece of learning content and provides details regarding how to use the selected learning content in his or her learning. For example, the learner can determine the period of studying and testing, the degree of rigor of the test, and the name of the scheduled learning.

**Fig. 6:**
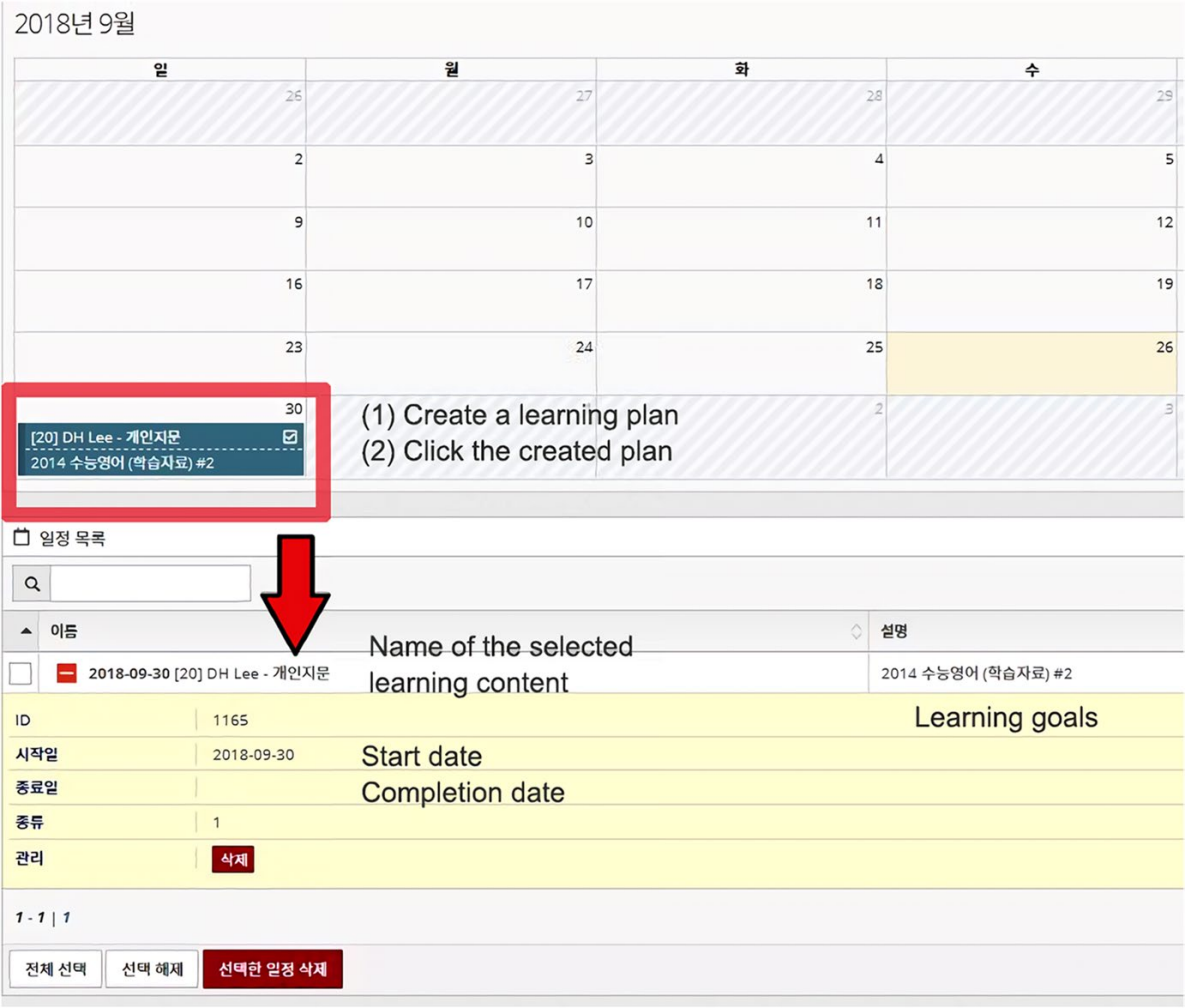
Screenshot of learning status

After the learning schedule is set, the management module displays information about the learner’s learning status, such as learning goals, duration, and achievements (Fig. 6), with a calendar interface. The information is automatically updated based on the learner’s activities.

**Fig. 7:**
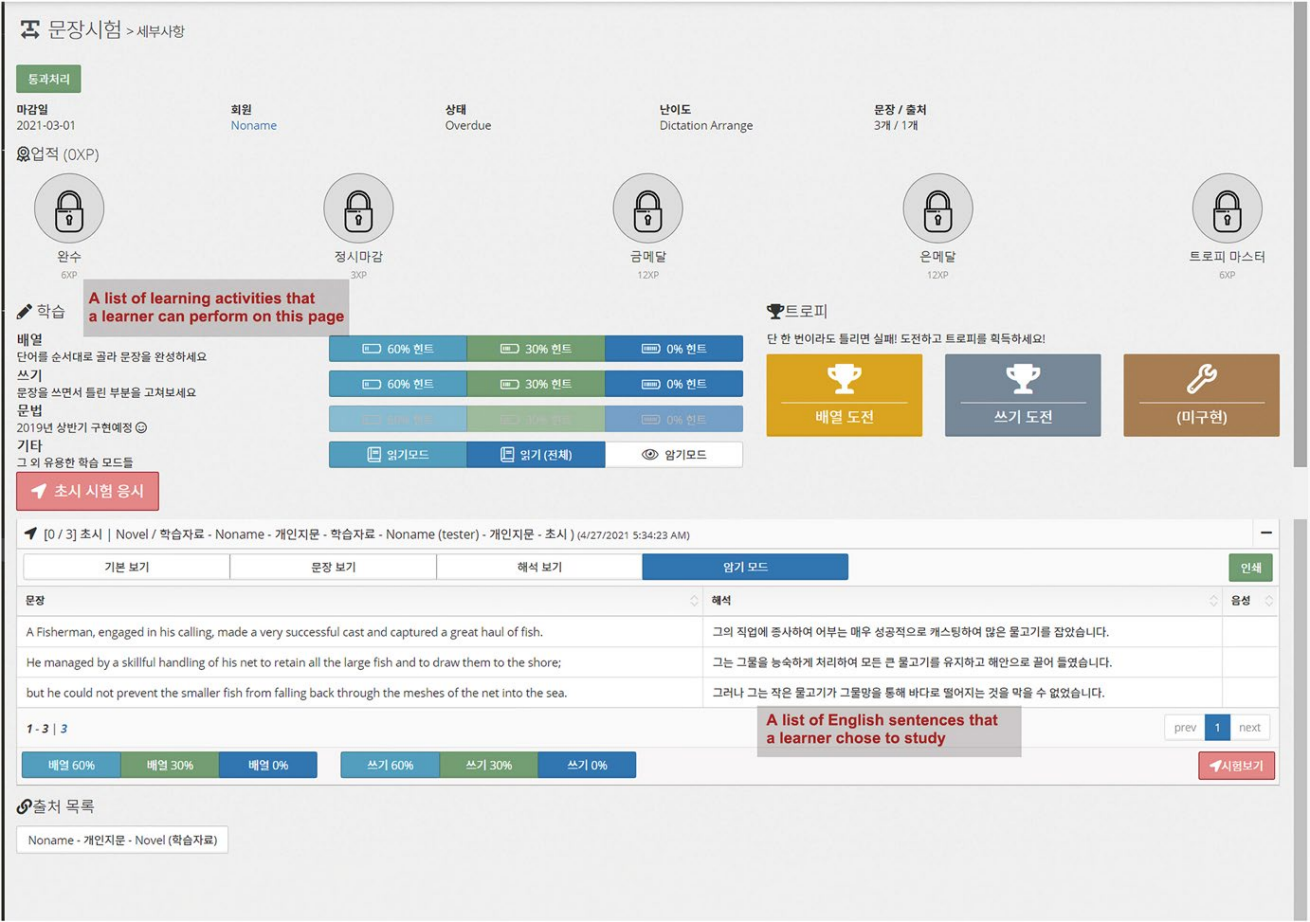
Screenshot of a page generated based on the data of the learning management module

Simultaneously, the personalized English language learning module scrapes the data generated in the learning management module and creates a page that allows the learner to carry out learning activities based on the data (Fig. 7). This function could strengthen LGC-based learning experiences because it does not use practice problems or tests from question banks pre-built by system developers or instructors. By clicking on a designated name in the learning schedule in the calendar-shaped user interface (UI), the learner moves to a page where he or she can conduct personalized practice or tests.

### Personalized English language learning module

The personalized English language learning module allows a learner to study English vocabulary or sentences using learning content selected by the learner. This module offers a “practice mode” and a “test mode,” in which the learner reviews and memorizes English vocabulary and sentences in a selected learning content and self-tests, respectively. The module operates in these two modes with a built-in intelligent agent that automatically generates sets of practice questions or tests by recognizing and analyzing the content that a learner selected. Table 1 summarizes the types of multi-sensory learning strategies and tasks a learner can perform in practice and test mode.

**Table 1:** Learning modes and corresponding activities offered by practice and test modes

Form of practice/test	Learning mode	Words and phrases learning mode	Sentences learning mode
Visualizing and typing activities	Practice	Flash cards with multiple choice, matching, and typing answers	Placing words in a sentence in the correct order
	Test	Quizzes (multiple choice; matching; short answer)	Placing words in a sentence in the correct order; filling out blanks in sentences
Listening activities	Practice	Dictation	Dictation
	Test	Dictation	Dictation
Speaking activities	Practice	–	Reading aloud; shadowing (listening to audio and recalling the sentence that the learner just listened to out loud, moving on to the next sentence, and repeating these steps)
	Test	–	Shadowing (listening to audio and recalling the sentence that the learner just listened to out loud, moving on to the next sentence, and repeating these steps)

**Fig. 8:**
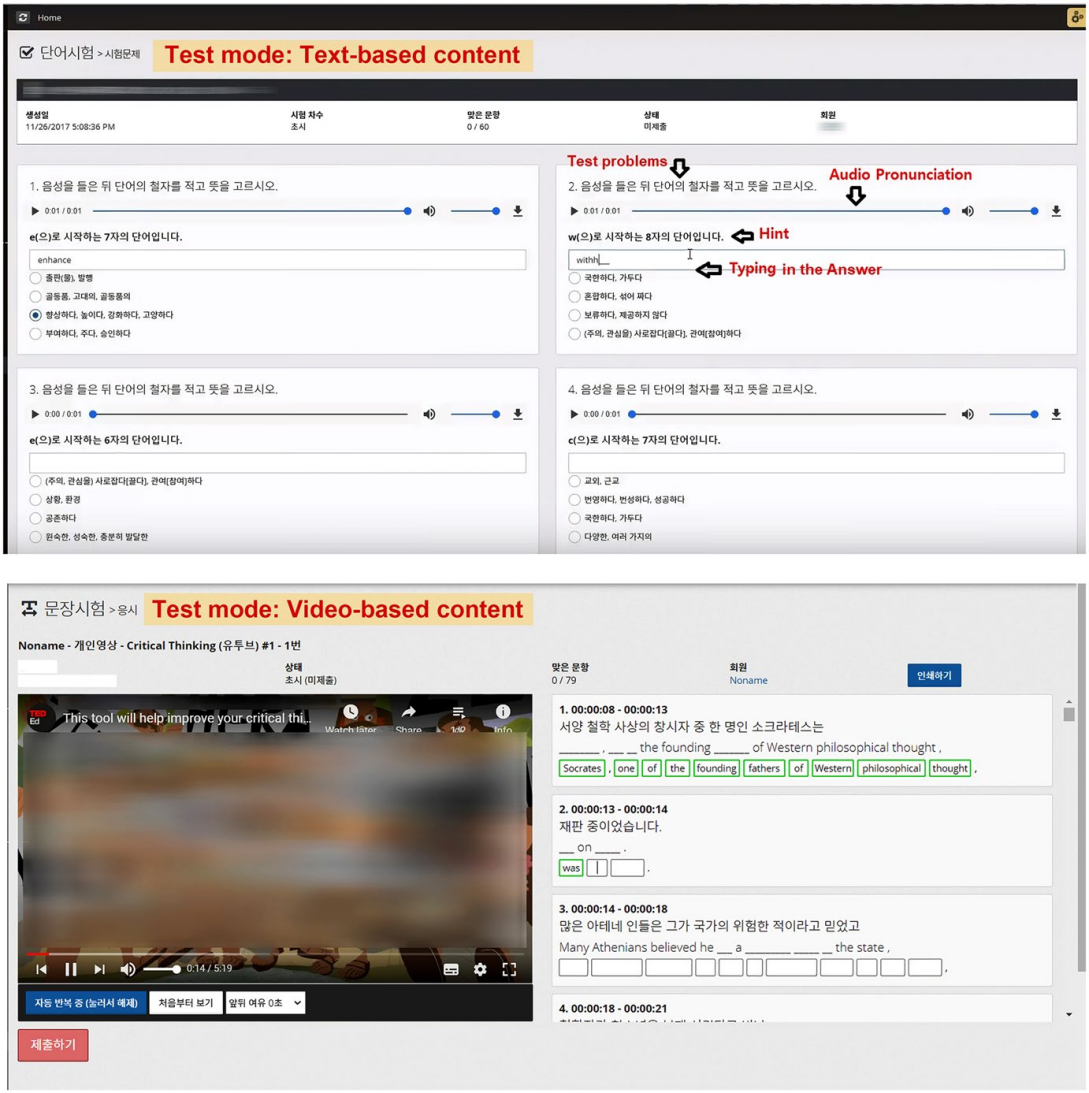
Test mode screen created by the personalized English language learning module based on the registered learning content

Figure 8 shows an example screen in test mode. Test scoring is done such that the module analyzes the answer that the learner inputs in the form of voice or text data and informs the learner of the accuracy of their answer between 0 and 100%.

The personalized English language learning module applies natural language processing APIs including text-to-speech and speech-to-text APIs, speech synthesis technologies. Our intention for this module was to immediately provide learners with the necessary information, stimulation, and feedback when performing multi-sensory activities such as speaking, listening, and writing according to individual learning style (scaffolding function). For example, by applying these APIs, the module generates data on the pronunciation of English text in the selected learning content and informs the learner how to pronounce the text. In addition, it can instantly measure and show the accuracy of the learner’s English pronunciation by recognizing the learner’s voice data when he or she records English-speaking activities using a microphone.

### Content curation module

**Fig. 9:**
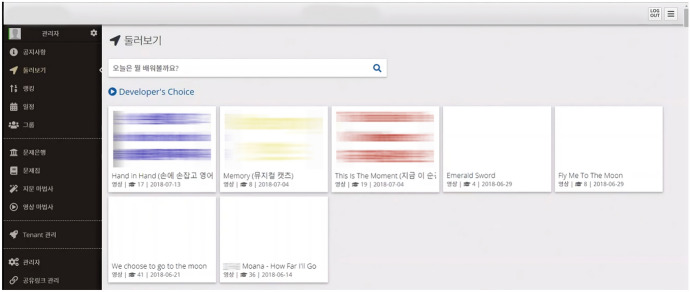
Learning content curation on the main page

The content curation module curates learner-generated content on the main page of the system (Fig. 9). With this module, the system’s main page can function as an open platform of content in which curated learning content in various subject areas and formats is exposed to multiple learners. Learners can continue learning by browsing or sharing content on the main page. The content curation module is connected to the learning content management module. Thus, the learners can import any curated content into the learning content management module and use them.

The functionality of the content curation module is limited because there was not enough data generated in the system for the module to learn its users' preferences. The curation module only has the function of automatically arranging content and displaying it on the main page. It sorts learning content firstly based on whether the content is text-based or video-based, and secondly, whether the content is for studying English words or sentences. Other meticulous work for curating content should be done by users by setting each content item.

This module does not have a content recommendation function. Instead, it allows the learner to view the profiles of learners who have created eye-catching content and to explore their other content.

## Evaluation of the system

### Field testing

To validate the developed system, we performed a field test of the system with actual English learners in Korea. This field test drew on our analyses of learners’ experiences. Learners form experiences through their interactions with the components of the digital learning environment (Kokoç & Altun, 2019), and it is possible to analyze those experiences by using data regarding their interactions with systems, interfaces, technologies, and content (Bouhnik & Marcus, 2006; Hillman et al., 1994; Wanstreet, 2006) or by collecting their own perceptions using digital learning tools and programs (Shi, 2014). Our field test examined whether the experiences learners formed by using our developed system reflected the characteristics of LGC-based learning.

### Recruitment of participants

The field test was approved by the Public Institutional Bioethics Committee designated by the Ministry of Health and Welfare of Korea. Participant recruitment and data collection and analysis were conducted between October and December 2020.

Three criteria were set for participant recruitment: resident of Seoul; secondary (middle or high) school student; and access to a digital device with Internet access. These criteria were chosen for the following three reasons. First, as mentioned earlier, Korean secondary school students have fewer opportunities to learn English with their own goals. Hence, they were identified as the top priority for the developed system. Second, because the system was only accessible online, the participants were required to have access to a device with Internet access, but we could not provide this. Finally, we attempted to prevent unnecessary long-distance travel by researchers and participants due to the COVID-19 pandemic.

The research participants were recruited online, and a field test was conducted with three secondary school students: Haru, Bada, and Sunny (pseudonyms). These students had varying educational backgrounds, and we believed that these participants would demonstrate different forms of learning experiences using the system.

### Data collection and analysis

After receiving signed consent forms from the participants and their legal guardians, data were collected through various activities to build an in-depth understanding of participants’ learning experiences using the system. The first activity was a tutorial lecture, an event presented to introduce the developed system and obtain preliminary survey information from the participants. Due to the possibility of COVID-19 resurgence, the participants were required to attend tutorial lectures on different dates (October 16, 17, and 21, 2020), and the tutorial was conducted in a large classroom in a building in Seoul. To ensure that all participants received consistent information on the system, the tutorial lectures were presenting following a pre-made manual. However, the special elements of each tutorial, such as student questions and opinions and researcher-student interactions, were recorded as textual observational data.

During the tutorial, participants were asked to complete a preliminary survey. This survey gathered demographic data as well as their perceptions of their previous English learning experiences and digitally supported English learning. The quantitative questions in this survey were scored on a 5-point Likert scale, ranging from 1 (*strongly disagree*) to 5 (*strongly agree*). After completing the survey, the students were provided with information on the functions of the system and introduced to sites offering OERs (e.g., open-access digital English textbooks and novels) and official YouTube channels related to movies and music so that they could reference when creating learning content. Approximately 30 samples of textual and video-based learning content created and curated by researchers were also introduced, and the students were advised to reuse the samples if they found it difficult to create learning content.

After the tutorial, the study participants were asked to freely perform English learning using the system over a seven-day trial period. To avoid influencing participants’ learning decisions, we did not intervene during this period. The participants were instructed to initiate contact with a researcher themselves if they required assistance.

Throughout the trial period, observational data were collected that included students’ learning content, information on their learning schedules and progress as recorded and displayed by the learning management module, and text data that recorded students’ actions, thoughts, and interactions.

After the trial period, student responses to online surveys and transcribed audio recordings of phone interviews were collected. The online survey included quantitative and qualitative items. The quantitative items covered topics such as use of and satisfaction with the developed system according to its function and the overall satisfaction with the system. These questions were scored on a 5-point Likert scale ranging from 1 (*strongly disagree*) to 5 (*strongly agree*). The qualitative items addressed the digital devices used to access the system, the learning locations, the person responsible for making decisions about the participants’ English language learning, the descriptions of participant behavior when using the system, the learning strategies applied, and opinions or identified problems regarding learning how to use the system. From the telephone interviews, students’ specific statements in response to questions raised from their survey responses or the researcher’s observational records were collected.

Table 2 summarizes the data collection procedure.

**Table 2:** Data collection

Participant	Data collection schedule
1. Haru	• Acquisition of consent, preliminary survey, and tutorial: October 21, 2020 • One-week developed system experience: October 22–28, 2020 • Learner experience survey (online): November 3, 2020 • Interview: November 14, 2020
2. Bada	• Acquisition of consent, preliminary survey, and tutorial: October 16, 2020 • One-week developed system experience: October 27–November 2, 2020 (postponed due to school examinations) • Learner experience survey (online): November 3, 2020 • Interview: November 13, 2020
3. Sunny	• Acquisition of consent, preliminary survey, and tutorial: October 17, 2020 • One-week developed system experience: October 30–November 5, 2020 (postponed due to school examinations) • Learner experience survey (online): November 9, 2020 • Interview: November 14, 2020

The data were analyzed in four stages using qualitative analysis strategies. First, by reading the data repeatedly, a holistic understanding of the English learning experience of each participant as it formed through the interactions with the system was constructed. Subsequently, specific or meaningful statements on participants’ activities and thoughts were extracted from the data (Ayres et al., 2003; Daher et al., 2017). Quantitative data that were collected through the survey were analyzed not for statistical verification but for the purpose of describing participants’ experiences (e.g., perceived satisfaction with the system, degree of use by function). All data were reviewed by cross-checking and identifying contradictory content, thereby building a coherent understanding of the learners’ experiences.

Second, based on the understanding of learners’ experiences, a narrative structure was constructed to describe individual learners’ experiences. The statements extracted in the previous stage were cited within the narrative structure.

Third, referring to the narrative descriptions of learners’ experiences, we examined whether their experiences implied the characteristics of LGC-based learning experiences, and if so, how, by associating them with the learning auxiliary elements (or the four modules) of the system. This work was guided by the three criteria of the main characteristics of LGC-based learning experience, consistent with our design principles, as well as one criterion for seeking problems with the system that would undermine the quality of the learner’s experience (see Table 3 for details).

**Table 3:** Criteria for judging the formation of LGC-based learning experiences through the system

Criterion 1	Could the learner access the developed system, set learning goals and content according to their needs, and begin learning even if the instructor or researcher did not pre-determine the learning context?
Criterion 2	Could learners interact with the four functional modules of the developed system and perform a series of associated learning activities, including generating learning content, scheduling learning, conducting practice and self-tests, and exploring others’ learning content?
Criterion 3	Could students further develop their learning experience by creating new ways of learning, exploring new learning content that they had not previously experienced, or communicating with other learners?
Criterion 4	Were any problems with the system reported that negatively affected the learner’s learning experience?

The analyses of the narrative descriptions were compared and discussed together. The unique properties of each experience were identified, along with the properties that were shared with other experiences (Miles et al., 2020). The following sections present the results of the field test.

### Introduction of participants

We first present an overview of the profiles of the student participants, including demographic information and their scores for their past English learning experiences and digital learning familiarity (Table 4), as well as their scores for the degree to which they used the system and their satisfaction during the 7-day trial period (Tables 5, 6).

**Table 4:** Participant profiles

	Haru	Bada	Sunny
[1] Gender	M	M	F
[2] Gender, grade, and school	2nd year of public middle school in Gyeonggi-Do surrounding Seoul	2nd year of public middle school in Seoul	2nd year of autonomous private high school^a^ in Gyeonggi-Do
[3] English learning experiences and thoughts so far			
1. How long have you been studying English so far?	Less than 1 year	More than 5 years	More than 5 years
2. Where did you usually study English?	Home	School	Private institute
3. How would you describe the main form of your English learning so far? (2 or more can be selected)	Personally decide on the learning content, make a plan, and then study English	Take a class from a private institute or extracurricular teacher and study English with the materials and assignments presented here	Take a class from a private institute or extracurricular teacher and study English with the materials and assignments presented here
4. What resources have you mainly used in your English learning? (2 or more can be selected)	Preparation book for national college entrance exam	School textbooks or school exam-relevant materials; Newspapers, magazines, and news in English	National college entrance exam preparation problem book; school textbooks or school exam-relevant materials; materials about TOEIC, TOEFL, etc
5. Who has mainly decided on the selection of learning materials and the learning methods in learning English?	Parents	Private institute, tutor	Private institute, tutor
6. Have your interests or opinions been sufficiently reflected in your learning content, plan, or strategy in English learning you have been doing so far?	4	2	2
7. Are you confident in your English skills?	1	4	3
[4] Thoughts on digitally based English learning			
Do you think learning English using online learning software or online learning aid systems will help you?	5	5	4
[5] Self- awareness of proficiency in digitally based English learning			
1. I can use the Internet for the purpose of studying English	4	4	5
2. I can find English learning information or content on web services such as YouTube, Internet news, cafes, and blogs	5	5	5
3. I can directly “manage” the information and content related to English learning that I find online	2	3	4
4. I can study English using digital devices with online access (e.g., PC, smartphone, tablet PC, notebook computer)	5	5	5
5. I can study English using online services (e.g., digital English learning apps, educational software, English learning blogs, YouTube)	5	5	4
6. I can study English using digital devices or artificial intelligence features (e.g., voice recognition, translator) provided by online services	5	5	5

^a^A type of private school in Korea that is financially independent of the government and is granted freedom in student selection and curriculum development

**Table 5:** Participants’ utilization scores

	Haru	Bada	Sunny
[1] Creation of learning materials			
1. I used the passage wizard to make the selected text and image materials into learning content	5	1	5
2. I used the video wizard to make the video material I selected as a learning content	3	5	5
3. I found other pieces of learning content that had been uploaded to the system and adjusted them again with the wizard to make them my learning content	1	1	1
[2] Calendar: Plan and manage learning			
1. I developed a learning schedule by selecting the learning content, date, and practice mode myself	3	4	5
2. I began learning by finding the personal learning page created according to the learning schedule	4	5	4
3. I checked the progress of my learning by checking the information displayed on the learning management page	2	5	3
[3] Personal study (practice, test)			
1. I studied the learning content I chose through the “practice” mode	5	2	2
2. I evaluated the status of my knowledge through the “test” mode	2	5	5
3. I achieved my English learning goals by using the practice mode and test mode	3	4	4
[4] Content curation function			
1. I shared the content I created on the main page	1	5	5
2. On the main page, I explored the pieces of learning content curated by other students	3	5	5

**Table 6:** Satisfaction scores

		Haru	Bada	Sunny
Overall satisfaction with the learning experience				
The English learning experience using the introduced system was generally satisfactory		4	5	4
Satisfaction by functional module				
[1] Learning content management module	Overall, the wizard function of creating learning content was satisfactory	4	5	4
[2] Learning management module	Overall, the ability to create and manage learning plans was satisfactory	5	5	3
[3] Personalized English language learning module	Overall, the practice mode was satisfactory	5	3	3
	Overall, the test mode was satisfactory	3	5	4
[4] Content curation module	Overall, the curation of the main page where I searched for content or shared my content was satisfactory	4	5	4

### Haru’s experience

#### Background

Haru is a male student at a general middle school located in the northern part of Gyeonggi-do who was staying in the Seoul area to attend classes at a private educational institute. According to his answers on the preliminary survey, he had the least English learning experience among the three participants (less than a year) and showed the lowest confidence in his English proficiency (1 point). He had studied alone at home while his parents decided on the general aspects of his English learning, such as the method of learning English and the selection of learning materials. However, he responded that his interests and opinions had been sufficiently reflected when studying English because his parents respected them.

He expressed low confidence in the management of information and content related to English learning on the Internet (2 points), but he answered that he was able to use the Internet to learn English (4 points) and that he was able to use various digital devices or online services to find content (5 points).

#### During the tutorial

He quietly listened to the tutorial lecture for an hour. A researcher asked him what his interests were. He responded, “For now, I don’t remember what I really want to use when I study.” He then added that he had been studying English by memorizing English vocabulary. During the tutorial, he did not show any noticeable reaction. However, he familiarized himself with the features of the system one by one.

#### During the seven-day trial period

It became apparent that Haru had clear ideas about learning English with the developed system. According to the interview, he thought that “this [the system] is definitely for studying English vocabulary.” Accordingly, he created several English vocabulary lists using the passage wizard and studied them for seven days.

When creating the English vocabulary lists, he first took some pictures of parts of the English textbooks or workbooks used in his school and uploaded them to the passage wizard, then sorted the English words he wanted to learn. Regarding this activity, he commented that the process was sometimes slightly inconvenient because the performance of the passage wizard’s OCR depends on the picture quality. Nevertheless, he said he was able to create vocabulary lists and study them without further difficulties: “I just did it because I set the learning goal and the study schedule without any special effort” (his answer to the online survey).

Interestingly, when studying the English vocabulary list, he found the practice mode to be more effective for memorizing content than the test mode and, therefore, rarely used the test mode (2 points for utility and 3 points for satisfaction with the test mode), instead mainly using the practice mode (5 points for utility and 5 points for satisfaction with the practice mode).

In the online survey and telephone interview, he pointed out three aspects he particularly liked about learning English using the system. First, he could have more English vocabulary lists than the school gave him. Second, he was able to study English vocabulary anytime and anywhere by accessing the system with his smartphone. Third, he stated that “[it] was good to be able to test your own knowledge by yourself.” In his view, the self-testing was convenient with this system because the module created and provided the test questions based on his learning content and instantly measured his answers’ accuracy.

However, as was previously mentioned, he had less than a year of experience learning English and had relied on one learning strategy, which was focused on memorizing English words. Even during the seven-day trial period, he used the system in a manner that made it easier to memorize English words. He had once attempted to generate video-based learning content but did not obtain satisfactory results.

Even if he relied on a single learning strategy, according to our assumptions, Haru should be able to take note of other possible learning activities by exploring the content and other learners’ activities through the content curation module. However, the module did not contribute to his exploratory activities. According to the online survey, Haru thought that the content curation function itself was good (4 points for satisfaction with the content curation module), but he did not share his own content or use other learners’ content curated on the main page (1 point for utility of content sharing; 3 points for utility of content exploration).

Several factors were revealed that influenced this low utility score for content curation. First, in contrast to the other two participants who delayed the seven-day trial period due to school exams, Haru began using the system as soon as the tutorial was over. Hence, he had little opportunity to explore content created by the other two participants.

Second, the content curation module was not able to function as a medium to spark his interest in video-based learning content or other learning strategies except memorizing English vocabulary because the volume and diversity of learning content curated by the module were insufficient.

Third, utilization of the content curation module may be low depending on the learner’s personal disposition. An incident occurred during Haru’s trial period in which he deleted all of his learning schedules and content registered in the system. In the interview, he stated, “I have finished studying all the English vocabulary [planned]” and “I deleted all the content. […] There was a desire to do something new with a new feeling.” That is, after completing his learning plans, he saw that the content (i.e., his English vocabulary lists) was no longer useful and eventually deleted the content he had made up to that point. In the process, it did not occur to him that there was a possibility of sharing his vocabulary lists with other learners.

This event revealed the possibility of a conflict between the personal tendencies of the original creator of the content and the content-sharing activity. If learners want to delete their own content or block the possibility of sharing based on their ownership of their content, how should this be handled? Active content sharing is important for LGC-based learning experiences, but it is also necessary to recognize the right to delete their own content to some extent. Haru’s experience suggests that additional measures are required to balance the guarantee of creators’ rights over their learning contents with the encouragement to share content within the system.

### Bada’s experience

#### Background

Bada is a male student attending a public middle school located in Seoul. In the preliminary survey and on-site conversation during the tutorial lecture, he stated that he had been studying English for more than five years and studied three times a week (three hours per session), mainly in private institute classes or private tutoring using the materials and assignments presented by the teacher. He had used various kinds of materials to study English, but primarily school textbooks, school exam preparation reference books, and news articles. Thanks to this long-term English learning experience, he stated that he was confident in his English skills. However, he also commented that his interests or opinions were hardly reflected in his learning experiences to date and that he mainly followed the opinions of academy instructors or private tutors. In the survey, he stated that he possessed skills related to web-based English learning and had “high expectations” for learning English using digital technology.

#### During the tutorial

In the tutorial lecture, he quietly listened to an introductory presentation of the developed system. Because he stated that he mainly studied English with an English problem book or textbook, the researcher expected that he would be interested in the OCR function of the passage wizard. However, he was considerably more interested in the video wizard. He mentioned his favorite fantasy movie and stated that he wanted to make learning material out of movie clips. At the end of the tutorial, Bada said that he was not sure what he expected from the system and added, “I think I can use the system for seven days and just figure it out” and “If I find anything difficult while trying it, I will seek help from a researcher or my college student sister.”

#### During the seven-day trial period

During the trial period, Bada accessed the system using a PC and studied English by establishing one to two learning schedules every two days. Notably, he never used the passage wizard at all. Instead, Bada pursued the interests he revealed in the tutorial: He created English learning content with YouTube videos and used them for his learning. He developed six video-based pieces of learning content over the course of seven days and learned 161 English sentences and 81 English words using them. Three of his learning materials were made from videos he found himself, and the other three were made by editing the sample content curated on the main page that the researchers uploaded during the tutorial (see Table 7 for details).

**Table 7:** Bada’s learning content and learning activity (Oct 27–Nov 2, 2020)

Source of learning content	Number of English words learned	Number of sentences learned	Explanatory note
Fantasy movie clip from official YouTube channel	131	34	Studied sample content curated on the main page
Science fiction movie clip from official YouTube channel	9	10	Studied content created by himself from a video that he chose
Animation movie clip from official YouTube channel	24	55	Studied sample content curated on the main page
Music video from official YouTube channel	19	47	Studied sample content curated on the main page
Music video from official YouTube channel	30	72	Studied content created by himself from a video that he chose
Music video from official YouTube channel	36	64	Studied content created by himself from a video that he chose

According to the interview, the process of creating video-based learning content was not smooth from the beginning. When he first attempted to create learning content using the video provided by his school teacher, he failed. However, he commented that the memory of such a failure was the most memorable activity: After the failure, he created video-based learning content with the help of his sister. Next, he successfully created video-based learning content by himself using movies, animations, and music videos and became proficient in content creation and independent learning using the content.

In the interview, two reasons were also suggested regarding how he was able to consistently create and study content using videos. First, he found the learning content management and learning management modules easy to use. Second, studying with the videos he liked became a motivation for learning, and he was thus able to study English alone “without much special effort” (his answer to the online survey). His answers in the online survey consistently reflected his thoughts. He gave 4–5 points for his utilization and satisfaction scores for the video wizard in the learning content management module.

However, his thoughts on the personalized English language learning module were varied. In the survey, he gave 2 points and 5 points for his utilization scores of the practice mode and test mode, respectively. This score resulted from his learning strategy: He found that taking the test multiple times was more effective for learning English vocabulary because scoring and feedback came quickly in test mode. It could therefore be used as a personalized workbook, which was sufficient to substitute for the practice mode. Therefore, after creating a piece of English learning content, Bada immediately took the test based on that content multiple times. When he judged that he had acquired enough knowledge from that content, he moved on, creating more learning content and a new learning schedule.

Regarding the content curation module, Bada gave a utilization score of 5 points. For seven days, he explored other content curated on the main page and selected three items of video-based content, with some adjustments, as his new learning content. He was also able to curate the content he created on the main page and thought positively about sharing his content: “I am proud of the idea that other students are studying with my learning content” (his answer to the online survey).

Bada rated his overall satisfaction with his English learning experience using the system as 5 points. He even recommended his own English learning strategy, which can be used in combination with the system: “Analyzing the lyrics of the English pop songs separately, applying the analysis results to the English learning content creation, and studying them” (his response in the interview).

Overall, Bada knew to seek others’ help when necessary, and once he became accustomed to a difficult task, he could control the overall learning process, including creating desired learning content, planning, acquiring knowledge from learning content, and engaging in self-assessment. He also invented his own learning strategy. Bada’s experience showed an outcome related to the personalized learning activity based on his LGC and an expansion of his learning context, which meets the criteria for LGC-based learning.

### Sunny’s experience

#### Background

Sunny is a high school student with a strong interest in attending university. Sunny’s school is an autonomous private high school that has been actively encouraging its students to attend excellent universities in Korea and abroad. Sunny intended to enter college with a computer science major. Through some programs offered by her school, she participated in various extracurricular activities related to the IT field. In the preliminary survey, she stated that she had sufficient ability to learn English using digital devices and software.

In the preliminary survey, she rated her English skills as 3 points. Although she had more than five years’ English learning experience, she thought that her interests and opinions had not been reflected in her learning process so far. Based on her perceptions of her previous English learning experiences, she described her expectations for the system as follows: “I hope that a ‘personalized’ learning curriculum that fits my level rather than a generalized class like a school is provided” (her statement during the tutorial).

#### During the tutorial

Sunny actively expressed her opinion during the tutorial lecture. Whenever she learned about one of the system’s functions, she immediately expressed agreement, liking the function, and other opinions. For example, she conveyed interest in the practice and test modes offering multi-sensory learning activities and requested an improvement of the UIs linking the learning content management and learning management modules because, in her view, it looked difficult to immediately find a page to schedule learning after making learning content.

#### During the 7-day trial period

During the 7 days of the trial period, Sunny stated that she used the system when she had 20 to 30 min of free time, such as during lunch breaks at school. At first, she tried the system with a variety of digital devices, but after finding that some functions of the system were excessively complex to use with a smartphone (e.g., the video and passage wizards), she decided to use the system with a laptop.

**Fig. 10:**
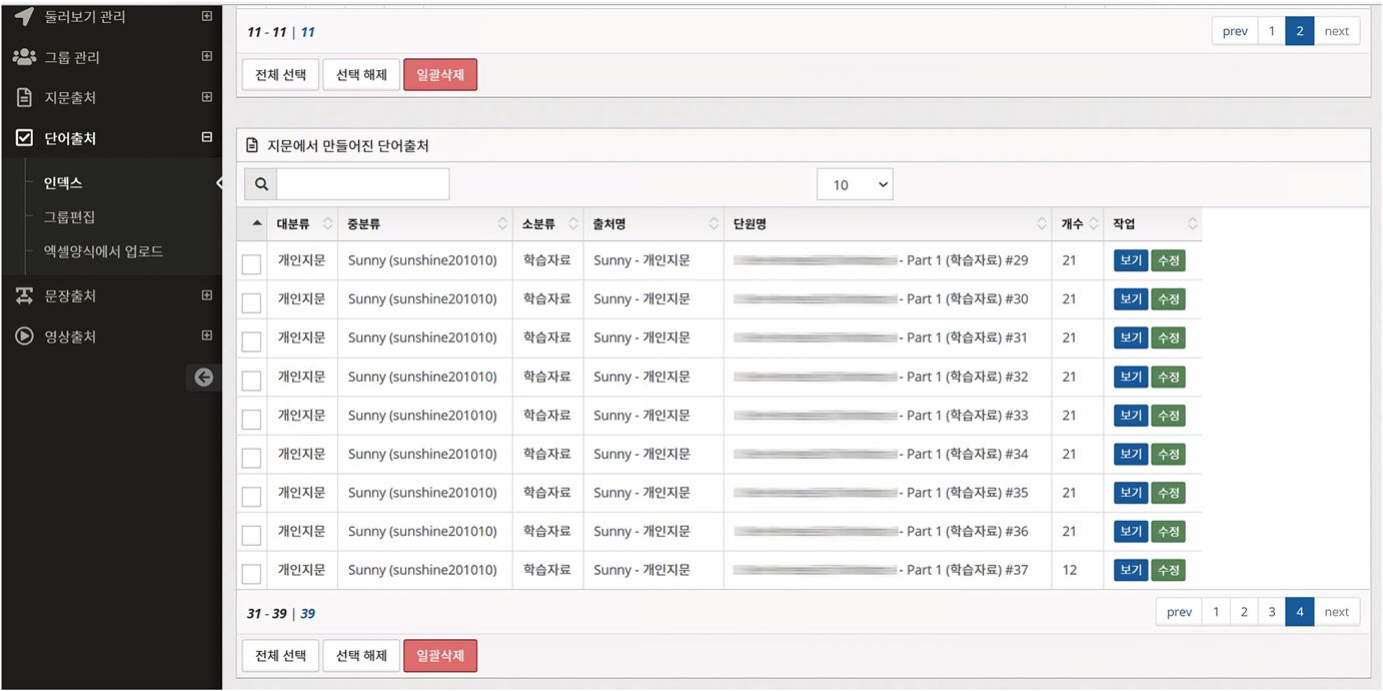
Records of Sunny’s learning content generated by the passage wizard

Sunny used both the passage and video wizards (5 points for her utilization of both the passage and video wizards). She first uploaded an A4 three-page English essay handout provided by the school to the passage wizard and then extracted the English text using OCR to create 37 English vocabulary lists. Each vocabulary list had a minimum of three and a maximum of 20 English vocabulary words, and by using them, she learned a total of 729 English words (see Fig. 10). Using the video wizard, she sorted the caption and voice data from the embedded official YouTube music videos and created video-based learning content with 40 English words and 45 English sentences.

According to the online survey and interview, using the learning content she created with the assistance of the system, Sunny developed a clear picture of what and how to learn from which pieces of content. After creating various pieces of learning content, she established learning schedules using the functions of the learning management module (5 points for her utilization of the function to set a learning schedule; 4 points for her utilization of the function to begin learning from the schedule) and performed learning activities with the created content using the functions of the personalized English language learning module (2 points for her utilization of the practice mode; 5 points for her utilization of the test mode). Here, similarly to Bada, Sunny studied the content by taking multiple tests and, therefore, gave a low score for her utilization of the practice mode. She also curated her content and explored other content (5 points for her utilization of the content-sharing and exploring activities) using the content curation module.

Throughout this process of studying the English language with the system, she conceived ideas for how to learn English using the system and delivered them to a researcher:

I felt that [this system] was a convenient learning aid when I was studying alone. […] I was able to learn words without searching for [the] meaning of the words one by one and even take a test by myself. Also, through various practice activities, I was able to read [English text] in a more interesting way than simply reading the printed text. […] I also felt that it was an advantage to be able to learn using new mediums that are not classic, such as English drama and TED-Ed videos. […] In terms of inducing individual interest, YouTube materials were good. (Her response in the interview)

Based on this experience, she recommended a strategy to study a single long English article (e.g., essay, passage from a novel, news article) to the researcher. This approach involved making some lists of English sentences from the article and doing the activities to peruse and learn the sentences, such as typing the words constituting each English sentence, shadowing the pronunciation of the sentences, or solving the test problems offered by the test mode. She said that this strategy would allow learners to carefully review the content of a single English article without missing its individual parts. She also added that this strategy was optimized for school exam preparation because most English tests in Korean schools use long English articles to develop test questions.

Meanwhile, based on her experience, she found that the current system had some problems, including a technical issue and a lack of curated content, which limited the personalized learning aids. During the trial period, she used a variety of the system’s features, such as creating content, setting a schedule for learning content, engaging in learning activities, and exploring curated content. However, what she wanted most during the period was to use TV news videos as learning content rather than music videos or movie clips, and the system failed to satisfy this interest. Video-based learning content creation using the video wizard was only easy when the video had caption data, and in contrast to music videos and movie clips, it was difficult for students to find news videos that provide captions. Using YouTube’s auto-captioning function, she attempted to create caption data by herself, but because the automated captions were not accurate, she could not make news-video–based learning content. This issue revealed the technical limitations of the developed system, suggesting the necessity of technical supplementation of the video wizard.

Because Sunny was concerned with university entrance examinations, she also wanted learning content that would help improve her academic grades, but she was disappointed to learn that the content curated on the main page was not helpful for this purpose. To complement the content curation module, she suggested that peer groups, particularly “peers from same school,” needed to use the system together and share content with each other.

As demonstrated by Sunny’s case, in order for the learner’s LGC-based learning experience to be sustainable, it is not only important to help learners study on their own from start to finish but also to facilitate active interactions with other learners or the exploration and sharing of varied content within the system. The issue that Sunny raised of facilitating learners’ active interactions and learner-generated content needs to be approached comprehensively, considering the issue of the “right of the original content creator” revealed in Haru’s case.

## Discussion

### Facilitation of LGC-based learning experience

In the field test, we found that the three learners’ experiences met the first two criteria for an LGC-based learning experience, and some met criterion 3 as well (criteria are listed in Table 4). Regarding criteria 1 and 2, although the learning context was not specifically defined, all three students were able to study English using the system. They pursued their own purposes and interests, created content, and conducted various learning activities to study English with that content. In this way, the students used the system to pursue novel interests (Bada) or existing interests in a more convenient way (Haru and Sunny). They also used the four system modules according to their own judgment and managed the overall aspects that constituted their learning experience, such as learning time, place, content, plan, and strategy. From criterion 3, some students devised new learning strategies with the system (Bada and Sunny).

Interestingly, the students occasionally used the system differently from our original purpose for it; for example, some used the test mode as a personal workbook rather than using the practice mode for this. Such selective uses did not hinder the formation of an LGC-based learning experience, as in both cases, the learner chooses how he or she will interact with the system. Altogether, all Korean participants experienced LGC-based learning while using the system to learn English.

However, what was less clear here was the influence of social conditions (or constraints) on learners’ context generation using the system. Sunny's experience showed that her self-determined learning goals were related to college entrance and school exams. According to Choi (2017), in Korean society, exams and test-rank competition are part of learners’ lives from an early age; accordingly, learners make choices to increase their chances of success on the exams. This indicates, even in the absence of specific instructions directing the participants’ choices, that some of the participants might have set the exam-focused goals preferred in the society as their own goals rather than exploring what they wanted to pursue by studying English. This issue may be a result of the extension of the learner-generated contexts being less supported by the developed system, which will be discussed in the next section. In addition, further research, including long-term field tests, will be required to assess this.

### Expanding learner-generated contexts

We developed a content curation module to assist learners in developing their learning contexts, but we found in field testing that participants did not substantially benefit from the module. Hence, additional measures are required to facilitate active interactions within the system.

First, we need to reinforce a supportive atmosphere for active content sharing and interaction among learners, and here, we consider two measures: adjusting the system’s content-sharing rule and developing individual communities of learners. Haru’s case offers an example of the need for the first change, adjusting the content-sharing rule: Students may be unfamiliar with sharing their learning content, and thus, we could allow content creators to delete their content at any time if the content has not yet been shared. Regarding the second idea, students with the same interests or educational background could be grouped within the content curation module function: learners could interact according to their common interests and help each other generate and share content.

Some measures that go beyond technical improvements are also required. In particular, we found that OERs could be useful for promoting active content sharing because there is not yet sufficient learner-generated content to meet learners’ diverse interests, OERs are ready made for education purposes, and they carry a lower risk of copyright infringement in relation to content use.

However, OERs have limited applicability to the current Korean context, namely, that it is difficult for secondary students to take advantage of these tools because OER-related sites in Korea are designed for use by higher education and secondary school instructors (Jung et al., 2011; Lim et al., 2019). We indirectly encountered this issue, where the participants created learning content with the resources were the most familiar to them and did not use the OER sites introduced in the tutorial at all. Optimizing OER use among students will therefore require increasing students’ familiarity with OERs and possibly developing more accommodating sites for young students.

### Need for human assistants

The field test revealed the importance of a human assistant who can support the interaction between students and the system, particularly managing the quality of content and encouraging learners to overcome their challenges. We observed this in the experience of Bada, who solved his difficulties creating learning content and materials with a sibling’s help and then performed English learning without difficulty. Incorporating a peer review mechanism into the system would allow learners to interact with other learners and be each other’s human assistants.

### Technological problems

We identified the following technological issues of the system: optimization and convenience problems with the passage and video wizards; difficulty operating some modules on a smart phone; and a nonintuitive UI for creating content and then a learning plan. To solve these issues, it is necessary to improve the performance of the wizards, develop a more mobile-friendly UI, and enhance the interface design of the learning management module.

In addition, YouTube's auto-captioning function did not accurately transcribe the English conversation in the video, which restricted students’ use of video-based learning content. It also seems again that the system can benefit from a human assistant who can help correct errors in automatically generated caption or text translation.

### Limitations of the research

This study had a limitation related to the characteristics of the field test participants, who, while they came from different backgrounds, all lived in urban areas in Seoul, were in secondary school, and had extensive knowledge of digital devices and services. This was partly because the conduct of the study was affected by the pandemic. In future research, it will be necessary to analyze the experiences of student participants representing a more diverse group to gain a more comprehensive understanding of the experience of Korean students using an LGC-informed, AI-based English learning support system.

## Conclusion

To borrow from Aiken and Epstein (2000), the challenge of AIEd research is to present how the philosophical premises of AI can respond to education needs, thereby not “limit[ing] the scope, effectiveness and positive contributions that AI can bring to learning” (p. 164). To address this challenge, we drew on the LGC framework and development research methodology, developed an AI-based English learning support system for Korean learners and examined whether and how the system could catalyze forming LGC-based learning experiences among the learners we studied. From our findings, we argue that an AI-applied learning assistance system based on sound educational technological design frameworks (specifically, in this study, the LGC framework) can catalyze learners’ autonomous learning experiences, even without a specified instructor, curriculum, or location, and help them become creators of their learning contexts. This study also provides a reference for AIEd researchers and practitioners pursuing similar goals.

We identified some issues regarding the developed system that we will need to address to enrich the LGC-based learning experience. These included the understanding of influence of social conditions and technical improvements, as well as enhancements in the educational environment and human assistants, as well as studies involving more diverse groups of learners. Our findings indicate that one AI learning support system alone cannot be the ultimate solution to LGC-based learning. However, according to Brandt (2013), tasting the freedom of learning itself can transform students’ thoughts on learning and encourages them to build ideas about their own learning paths. In this sense, we believe, by enabling such a tasting, one AI-applied tool could meaningfully contribute to education, “unleashing the innovative potential of students” (Ball, 2018, p. 235).

## Data Availability

The data of this study are not publicly available due to human subjects research protection agreed to in the informed consent process, under institutional review.

## References

[CR1] Aguayo, C., Cochrane, T., & Narayan, V. (2017). Key themes in mobile learning: Prospects for learner-generated learning through AR and VR. *Australasian Journal of Educational Technology*. 10.14742/ajet.3671

[CR2] Ahmad MF, Ghapar WRGWA (2019). The era of artificial intelligence in Malaysian higher education: Impact and challenges in tangible mixed-reality learning system toward self exploration education (SEE). Procedia Computer Science.

[CR3] Aiken RM, Epstein RG (2000). Ethical guidelines for AI in education: Starting a conversation. International Journal of Artificial Intelligence in Education.

[CR4] Ariebowo T (2021). Autonomous learning during COVID-19 pandemic: Students’ objectives and preferences. Journal of Foreign Language Teaching and Learning.

[CR5] Ayres L, Kavanaugh K, Knafl KA (2003). Within-case and across-case approaches to qualitative data analysis. Qualitative Health Research.

[CR6] Ball SJ (2018). The tragedy of state education in England: Reluctance, compromise and muddle—A system in disarray. Journal of the British Academy.

[CR7] Blaschke, L. M., & Hase, S. (2016). Heutagogy: A holistic framework for creating twenty-first-century self-determined learners. In *The future of ubiquitous learning* (pp. 25–40). Springer, Berlin, Heidelberg. 10.1007/978-3-662-47724-3_2

[CR8] Bouhnik D, Marcus T (2006). Interaction in distance-learning courses. Journal of the American Society for Information Science and Technology.

[CR9] Brandt B, Hase S, Kenyon C (2013). The learner’s perspective. Self-determined learning: Heutagogy in action.

[CR10] Brown, G. T. (2003). Teachers’ instructional conceptions: Assessment’s relationship to learning, teaching, curriculum, and teacher efficacy. In *joint conference of the Australian and New Zealand Associations for Research in Education (AARE/NZARE)* 28.

[CR11] Chang OH (2006). 학습자 자율성 신장을 위한 웹기반 자기접근식 영어 읽기학습: 고등학생 질적 사례연구 [Self-accessible web-based English reading for developing learner autonomy: Qualitative case study for high school students]. Multimedia-Assisted Language Learning.

[CR12] Choi S (2017). Schooling, learning disposition, and life course transitions: A life history study on Korean elite adult learners. Adult Education Quarterly.

[CR13] Cochrane T, Bateman R (2011). Strategies for mlearning integration: Evaluating a case study of staging and scaffolding mlearning integration across a three-year bachelor’s degree. Waikato Journal of Education.

[CR14] Cochrane, T., & Narayan, V. (2011). DeFrosting professional development: Reconceptualising teaching using social learning technologies. In D. Hawkridge, K. Ng & S. Verjans (Eds.), *Proceedings of ALT-C 2011 - Thriving in a colder and more challenging climate: The 18th international conference of the Association for Learning Technology* (pp. 158–169). ALT Association for Learning Technology, University of Leeds. 10.3402/rlt.v19s1/7796

[CR15] Cochrane, T., Antonczak, L., & Wagner, D. (2012). Heutagogial Approaches to mlearning: from Student-generated Content to International Co-production. In *Mlearn* (pp. 156–163).

[CR16] Conati, C. (2009). Intelligent tutoring systems: New challenges and directions. In *International joint conference on artificial intelligence* (Vol. 9, pp. 2–7). Pasadena, CA.

[CR17] Cook, J. (2010). Mobile learner generated contexts. In *Medienbildung in neuen Kulturraumen* (pp. 113–125). VS Verlag fur Sozialwissenschaften. 10.1007/978-3-531-92133-4_8

[CR18] Cronin C (2017). Openness and praxis: Exploring the use of open educational practices in higher education. The International Review of Research in Open and Distributed Learning.

[CR19] Daher M, Carré D, Jaramillo A, Olivares H, Tomicic A (2017). Experience and meaning in qualitative research: A conceptual review and a methodological device proposal. Forum Qualitative Social Research.

[CR20] Dignum V (2021). The role and challenges of education for responsible AI. London Review of Education.

[CR21] Djoub, Z. (2016). Mobile technology and learner autonomy in language learning. In *Human-Computer Interaction: Concepts, methodologies, tools, and applications* (pp. 291–309). IGI Global.

[CR22] Duffy, P., & Bruns, A. (2006). The use of blogs, wikis, and RSS in education: A conversation of possibilities. In Brown, A (Ed.), *Learning on the move: Proceedings of the online learning and teaching conference 2006* (pp. 31–38). Queensland University of Technology, CD Rom. Retrieved from https://eprints.qut.edu.au/5398/1/5398.pdf

[CR23] Fernández M, Wegerif R, Mercer N, Rojas-Drummond S (2015). Re-conceptualizing “scaffolding” and the zone of proximal development in the context of symmetrical collaborative learning. Journal of Classroom Interaction.

[CR24] Fryer LK, Coniam D, Carpenter R, Lăpușneanu D (2020). Bots for language learning now: Current and future directions. Language Learning & Technology.

[CR25] Hargreaves A (2021). What the Covid-19 pandemic has taught us about teachers and teaching. Facets.

[CR26] Hillman DCA, Willis DJ, Gunawardena CN (1994). Learner-interface interaction in distance education: An extension of contemporary models and strategies for practitioners. American Journal of Distance Education.

[CR27] Hyun J, Im H (2019). Analysis and implications of AI speakers as English learning tools. Journal of Mirae English Language and Literature.

[CR28] Inyega HN, Inyega JO (2020). Machine learning: The future of sustainable teacher education is here. Journal of Pedagogy, Andragogy and Heutagogy in Academic Practice/ISSN: 2708-261X.

[CR29] Jeon, J. (2010). Issues for English tests and assessments: A view from Korea. *Language assessment in Asia: Local, regional or global*, 55–82.

[CR30] Jung, H. J., Kwon, S. J., Cho, H.Ch., & Kim, D. S. (2011). 국내 OER 현황 분석 및 발전방향 탐색 [Study on the problems and improvement directions of Open Educational Resource systems]. In *Proceedings of the Korea Contents Association Conference* (pp. 223–224). The Korea Contents Association.

[CR31] Juřičková R (2013). Recognizing personal learning styles and using learning strategies while learning English in an electronic environment. Journal on Efficiency and Responsibility in Education and Science.

[CR32] Kim H, Shin D, Yang H, Lee JH (2019). A study of AI chatbot as an assistant tool for school English curriculum. Journal of Learner-Centered Curriculum and Instruction.

[CR33] Kim, H. J., & Won, H. H. (2019). 의사소통 능력 향상을 위한 영어교육 개선방안 모색: 덴마크와의 비교를 중심으로 [Search for improvement of English education system to enhance communication ability: Focusing on the comparison with Denmark]. *Journal of Fisheries and Marine Sciences Education, 31*(4), 1009–1020. 10.13000/JFMSE.2019.8.31.4.1009

[CR34] Kim, H. W., Choi, H. J., Kim, N. R., Shin, J. E., Shin, E. S., Cheong, M. S., ... Ahn, Y. E. (2020). *코로나19가 교사의 수업**, **학생의 학습 및 가정생활에 미친 영향**: **중**, **고등학교를 대상으로 [The impact of COVID-19 on teachers' classrooms, students' learning and family life: Focused on middle and high schools]* (Report No. 서교연 2020–59). Seoul Education Research & Information Institute. https://www.serii.re.kr/fus/file/down0010f.do?attach_file_id=4417&file_seq=1&alias_name=20210830033851585_1

[CR35] Kokoç, M., & Altun, A. (2019). Building a learning experience: What do learners’ online interaction data imply?. In *Learning technologies for transforming large-scale teaching, learning, and assessment* (pp. 55–70). Springer, Cham. 10.1007/978-3-030-15130-0_4

[CR36] Korea Education and Research Information Service. (2020). *Covid-19에 따른 초, 중등학교 원격교육 경험 및 인식 분석 [Analysis of the experiences and perceptions of distance education in elementary and secondary schools in the context of Covid-19 pandemic].* Korea Education and Research Information Service. Retrieved from https://www.keris.or.kr/main/ad/pblcte/selectPblcteETCInfo.do?mi=1142&pblcteSeq=13356

[CR37] Kukulska-Hulme A (2010). Mobile learning as a catalyst for change. Open Learning: THe Journal of Open, Distance and e-Learning.

[CR38] Kwon SK, Lee M, Shin D (2017). Educational assessment in the Republic of Korea: Lights and shadows of high-stake exam-based education system. Assessment in Education : Principles, Policy & Practice.

[CR39] Lai C (2019). Technology and learner autonomy: An argument in favor of the nexus of formal and informal language learning. Annual Review of Applied Linguistics.

[CR40] Lambert C, Philp J, Nakamura S (2017). Learner-generated content and engagement in second language task performance. Language Teaching Research.

[CR41] Lee MJW, McLoughlin C (2007). Teaching and learning in the Web 2.0 era: empowering students through learner-generated content. International Journal of Instructional Technology and Distance Learning.

[CR42] Lee SM (2020). The impact of using machine translation on EFL students’ writing. Computer Assisted Language Learning.

[CR43] Lee SM, Briggs N (2021). Effects of using machine translation to mediate the revision process of Korean university students’ academic writing. ReCALL.

[CR44] Lim KY, Lim SH, Jo IH, Lim JY, Han GY (2019). 멀티미디어 설계원리를 적용한 공개교육자료 사례 분석 및 학습자 인식 탐색 [Application of multimedia design principles: A case study of a video lecture for open educational resources]. The Journal of Learner-Centered Curriculum and Instruction.

[CR45] Loeckx J (2016). Blurring boundaries in education: Context and impact of MOOCs. International Review of Research in Open and Distributed Learning.

[CR46] Luckin R (2010). Learning contexts as ecologies of resources: A unifying approach to the interdisciplinary development of technology rich learning activities. International Journal on Advances in Life Sciences.

[CR47] Luckin, R., Akass, J., Cook, J., Day, P., Ecclesfield, N., Garnett, F., Gould, M., Hamiltion, T., & Whitworth, A. (2007). Learner-generated contexts: Sustainable learning pathways through open content. In P. McAndrew & J. Watts (Eds.), *Proceedings of the OpenLearn2007 conference* (pp. 90–94). Milton Keynes. Retrieved from http://citeseerx.ist.psu.edu/viewdoc/download?doi=10.1.1.557.3949&rep=rep1&type=pdf

[CR48] Luckin, R., Clark, W., Garnett, F., Whitworth, A., Akass, J., Cook, J., Day, P., Ecclesfield, N., Hamiltion, T., & Robertson, J. (2011). Learner-generated contexts: A framework to support the effective use of technology for learning. In *Web 2.0-based e-learning: Applying social informatics for tertiary teaching* (pp. 70–84). IGI Global.

[CR49] Luckin R, Cukurova M (2019). Designing educational technologies in the age of AI: A learning sciences-driven approach. British Journal of Educational Technology.

[CR50] Luckin, R., Holmes, W., Griffiths, M., & Forcier, L. B. (2016). *Intelligence unleashed: An argument for AI in education.* Pearson.

[CR51] Luckin, R., Puntambekar, S., Goodyear, P., Grabowski, B. L., Underwood, J., & Winters, N. (2013). The ecology of resources: A theoretically grounded framework for designing next generation technology-rich learning. In *Handbook of design in educational technology* (pp. 45–55). Routledge.

[CR52] Maru MG, Pikirang CC, Setiawan S, Oroh EZ, Pelenkahu N (2021). The internet use for autonomous learning during Covid-19 pandemic and its hindrances. International Journal of Interactive Mobile Technologies.

[CR53] McLoughlin C, Lee MJ (2008). The three p's of pedagogy for the networked society: Personalization, participation, and productivity. International Journal of Teaching and Learning in Higher Education.

[CR54] Miles, M. B., Huberman, A. M., & Saldaña, J. (2020). *Qualitative data analysis: A methods sourcebook*. Sage Publications.

[CR55] Narayan V, Davis C, Gee R (2012). Augmented learning–spreading your wings beyond the classroom. Research in Learning Technology.

[CR56] Narayan, V., & Herrington, J. (2014). Towards a theoretical mobile heutagogy framework. In B. Hegarty, J. McDonald, & S.-K. Loke (Eds.), *Rhetoric and reality: Critical perspectives on educational technology, Ascilite 2014* (pp. 150–160). Dunedin: Ascilite. Retrieved from http://researchrepository.murdoch.edu.au/26680/1/hautagogy_framework.pdf

[CR57] Narayan V, Herrington J, Cochrane T (2019). Design principles for heutagogical learning: Implementing student-determined learning with mobile and social media tools. Australasian Journal of Educational Technology.

[CR58] Oh YK (2022). 중학생 영어 학업성취도에 영향을 미치는 정의적 변인 간의 구조관계 분석 [A structural analysis of affective variables influencing middle school students’ English achievement]. The Journal of Curriculum and Evaluation.

[CR59] Organization for Economic Co-Operation and Development (2019). OECD skills outlook 2019: Thriving in a digital world. OECD Publishing.

[CR60] Ox J, Van Der Elst J (2011). How metaphor functions as a vehicle of thought: Creativity as a necessity for knowledge building and communication. Journal of Visual Art Practice.

[CR61] Pachler N, Bachmair B, Cook J (2009). User-generated content and contexts: An educational perspective. Mobile learning.

[CR62] Pachler N, Bachmair B, Cook J (2010). Mobile learning: Structures, agency, practices.

[CR63] Palalas A, Wark N (2020). A framework for enhancing mobile learner-determined language learning in authentic situational contexts. International Journal of Computer-Assisted Language Learning and Teaching (IJCALLT).

[CR64] Pantelimon FV, Bologa R, Toma A, Posedaru BS (2021). The evolution of AI-driven educational systems during the COVID-19 pandemic. Sustainability.

[CR65] Park J (2019). An AI-based English grammar checker vs. human raters in evaluating EFL learners’ writing. Multimedia-Assisted Language Learning.

[CR66] Park JH, Yang IY (2020). Utilizing an AI-based grammar checker in an EFL writing classroom. Korean Journal of Applied Linguistics.

[CR67] Ponerulappan P (2015). Content curation: a tool for stimulating the use of learning resources and social learning. ZENITH International Journal of Multidisciplinary Research.

[CR68] Rahimi E, van den Berg J, Veen W (2015). A learning model for enhancing the student's control in educational process using Web 2.0 personal learning environments. British Journal of Educational Technology.

[CR69] Richey RC, Klein JD (2005). Developmental research methods: Creating knowledge from instructional design and development practice. Journal of Computing in Higher Education.

[CR70] Sharpe, R. (2010). Conceptualizing differences in learners' experiences of e-learning: A review of contextual models. *Report of the Higher Education Academy LearnerDifference(HEALD) Synthesis Project.* Retrieved from http://www.heacademy.ac.uk/resources/detail/evidencenet/Conceptualizing_differences_in_learners_experiences_of_e-learning

[CR71] Shi, L. (2014). Defining and evaluating learner experience for social adaptive e-learning. In *OASIcsOpenAccess Series in Informatics* (pp. 74–82). Schloss Dagstuhl - Leibniz Center for Informatics. 10.13140/2.1.1720.2564

[CR72] Smrz, P. (2004). Integrating natural language processing into e-learning-A case of Czech. In *Proceedings of the workshop on eLearning for computational linguistics and computational linguistics for E-learning* (pp. 1–10).

[CR73] Vavoula, G. N., & Sharples, M. (2002). KLeOS: A personal, mobile, knowledge and learning organisation system. In *IEEE international workshop on**wireless and mobile technologies in education, 2002. Proceedings *(pp. 152–156). IEEE.

[CR74] Wanstreet CE (2006). Interaction in online learning environments: A review of the literature. Quarterly Review of Distance Education.

[CR75] Wismaningrum, Y.R., Prayitno, H.J., & Supriyanto, E. (2020). Heutagogy approach: The implementation of new normal era learning. In *The 5th progressive and fun education international conference (PFEIC 2020)* (pp. 189–193). Atlantis Press. 10.2991/assehr.k.201015.029

[CR76] Woo JH, Choi HY (2021). Systematic review for AI-based language learning tools. Journal of Digital Contents Society.

[CR77] Zawacki-Richter O, Marín VI, Bond M, Gouverneur F (2019). Systematic review of research on artificial intelligence applications in higher education–where are the educators?. International Journal of Educational Technology in Higher Education.

[CR78] Zhai X, Chu X, Chai CS, Jong MSY, Istenic A, Spector M, Liu J-B, Yuan J, Li Y (2021). A review of artificial intelligence (AI) in education from 2010 to 2020. Complexity.

[CR79] Zhang D, Nunamaker JF (2004). A natural language approach to content-based video indexing and retrieval for interactive e-learning. IEEE Transactions on Multimedia.

